# SARS-CoV-2 Bound Human Serum Albumin and Systemic Septic Shock

**DOI:** 10.3389/fcvm.2020.00153

**Published:** 2020-09-02

**Authors:** Andrew S. Johnson, Rouholah Fatemi, William Winlow

**Affiliations:** ^1^Dipartimento di Biologia, Università Degli Studi di Naples, Federico II, Naples, Italy; ^2^Physiology Research Center (PRC), School of Medicine, Ahvaz Jundishapur University of Medical Sciences, Ahvaz, Iran; ^3^Institute of Ageing and Chronic Diseases, The Apex Building, University of Liverpool, Liverpool, United Kingdom

**Keywords:** human serum albumin, septic shock, coronavirus, endothelial glycocalyx layer, acute respiratory distress syndrome, albumin therapy

## Abstract

The emergence of the COVID-19 virus and the subsequent pandemic have driven a great deal of research activity. The effects of COVID-19 are caused by the severe respiratory syndrome coronavirus 2 (SARS-CoV-2) and it is the underlying actions of SARs-CoV-2 virions on the endothelial glycocalyx that we consider here. One of the key factors in COVID-19 infection is its almost unique age-related profile, with a doubling in mortality every 10 years after the age of 50. The endothelial glycocalyx layer is essential in maintaining normal fluid homeostasis, but is fragile and prone to pathophysiological damage. It is physiologically significant in capillary microcirculation and in fluid distribution to the tissues. Human serum albumin (HSA), the most abundant protein in plasma, is created in the liver which also maintains its concentration, but this reduces by 10–15% after 50 years of age. HSA transports hormones, free fatty acids and maintains oncotic pressure, but SARS-CoV-2 virions bind competitively to HSA diminishing its normal transport function. Furthermore, hypoalbuminemia is frequently observed in patients with such conditions as diabetes, hypertension, and chronic heart failure, i.e., those most vulnerable to SARS-CoV-2 infection. Hypoalbuminemia, coagulopathy, and vascular disease have been linked in COVID-19 and have been shown to predict outcome independent of age and morbidity. Hypoalbuminemia is also known factor in sepsis and Acute respiratory distress syndrome (ARDS) occurs when fluids build-up in the alveoli and it is associated with sepsis, whose mechanism is systemic, being associated with the fluid and logistic mechanisms of the circulation. Glycocalyx damage is associated with changes plasma protein concentration, particularly HSA and blockage of albumin transport can produce the systemic symptoms seen in SARS-CoV-2 infection and sepsis. We therefore conclude that albumin binding to SARS-CoV-2 virions may inhibit the formation of the endothelial glycocalyx by inhibition of albumin transport binding sites. We postulate that albumin therapy to replace bound albumin might alleviate some of the symptoms leading to sepsis and that clinical trials to test this postulation should be initiated as a matter of urgency.

## Introduction

The COVID-19 pandemic has renewed interest in emergent pathogens as a major threat for human health. To date many millions have been infected and hundreds of thousands or greater mortality is expected. Quantitative approaches to the current outbreak are urgently needed to tackle this severe disease.

Coronaviruses are found in many different species of animals (e.g., bats and dromedaries) and can become infectious in humans. Typical spread is by droplets from coughing or sneezing, aerosol, or direct contamination for example through stools. The active virion SARS-CoV survives well and can survive without denaturing for up to 2 weeks in stools after infection. Coronavirus infections are not new, the Severe Acute Respiratory Syndrome (SARS-CoV-2), reported in Asia in February 2003 resulted in almost 9,000 cases with a case-fatality rate of 10%. In 2012, (MERS-CoV) infected more than 2,500 people, killing over 800.

Epidemiological models help policy makers to take decisions. Social distancing has helped mitigate the spread of the epidemic and lockdown of successive geopolitical zones has sequentially reduced both spread and mortality of the disease. Such methods could eradicate the virus but necessitate global co-operation, as does vaccination.

There are many epidemiological unknowns with COVID-19. Infection and mortality rates are still being collated from different sources with varying degrees of accuracy and reliability. Whilst much is uncertain in COVID-19 pandemic, aspects of the disease are well documented such as age related mortality effectively doubling for each 10 years after 50, obesity (1) and the effects on black, Asian and minority ethnic (BAME) individuals (2) which may be unrelated to direct environmental factors and for which there is some evidence of a link to melatonin (3).

One of the difficulties with research into the human body is the transposition of these presentations to cellular physiological and molecular causative events. Of greater mathematical complication is the necessity to understand how within a whole-body cellular infection caused by molecular events lead to systemic injury and death.

In this paper our attention is not in in predicting who dies, but in using this data to find out what corporeal elements have been systemically disturbed and which can account for the differences in mortality across aging, sex and obesity among others. The answer has to be found at the molecular level and as already suggested in the logistical supply of trophic materials to allow the virus to multiply safely without precipitating cell death before the inflection point where the speed of antibody production to remove the virus is enough not to precipitate sepsis. Pathology of systemic sepsis is associated with death in COVID-19 patients (4).

## Infection

Our understanding of the infection at the cellular level is still poor but we can make assumptions based upon peer-reviewed studies of other infections. Some direct experimentation has already taken place, for example the effects on age from experiments on macaques (5). However, presentation of death related factors and statistical evaluation at the population level of infection and subsequent death rates has resulted in some factors of universal agreement across many countries. A discussion of the exact nature and precision of these evaluations is beyond the scope of this paper, but enough trends from statistical evaluation have been corroborated that allow us to understand both the infection of single individuals and the passage of the COVID-19 virions as they infect single cells and multiply leading to systemic infection and sometimes death.

There have been many suggested criteria examined for prognosis of COVID-19 and these epidemiological markers have been used to selectively profile and isolate members of communities in order to offer protection. The most important of these is the almost unique presentation of age profile, which in epidemiological terms occurs after 50 years of age with a doubling in mortality every 10-years thereafter. Unlike during the 1918 influenza outbreak where prognosis for both the young and the old was one of the major factors of mortality n COVID-19 the majority of deaths are in the over 50s. This difference is crucial in defining the cellular and molecular actions of the virus in infection. Similarly, the high death rates for obese patients and BAME communities may give indications of the mechanism of the virus action as well as the need for protection of minorities. Symptoms of the disease are also indicators for the metabolic distribution of the virions around the body at different stages of the disease. Finally, it must be remembered that at all stages, death is usually presaged by systemic spread of the disease leading to sepsis, which is discussed in detail below. If we are to successfully model the cellular and subcellular of COVID-19, then all forms of systemic stress, such as the multi system inflammatory syndrome (MSIS) Kawasaki type illnesses seen in young children (6) and multi organ failure and sepsis, must be identified.

## Infection Vector

Primary presentation of COVID-19 can include shortness of breath, coughing, loss of smell and taste, and stomach pains, but may include many other symptoms. At the cellular level there are many common events that can be quantified. The infection vector is usually respiratory. SARS-CoV-2 binds to the common angiotensin-converting enzyme 2 (ACE2) (7) cell-surface receptor (8, 9). The virus infects cells using the ACE 2 receptor a precursor in the renin-angiotensin system to angiotensin II a vasoconstrictor. ACE 2 is present in many cell membranes of also all organs and serves numerous purposes within the cell. Infection usually occurs through the lungs, but the ACE 2 receptors are also represented throughout the intestine and nasal epithelia and can also take these routes (5).

ACE2 is prevalent throughout the body. Prevalence of ACE2 makes tissues vulnerable to infection, as the virus requires that receptor be present to enter a cell (7, 9, 10). Virion RNA is then released into the cell where it mimics the cells own messenger-RNA (mRNA). SARS-CoV-2 RNA impersonates the cell's own mRNA instructing the cell to produce the relevant proteins to construct new virions which are then released from the cell. Antiviral drugs act against this mechanism. The ability of antiviral drugs to slow virion multiplication is an important element against the initial infection. However, deaths ascribed are not due to the SARS-CoV-2 virion but to the consequent disruption to cellular components—this is evidenced by the difference between old and young victims. In older individuals, changing metabolism has rendered them vulnerable. The source of this disruption may only partly be due to the virion itself.

In COVID-19, infection manifests in the alveoli of the upper lungs. Oxygen crosses the alveoli into tiny capillaries that lie beside the air sacs (11, 12). The oxygen is then carried to the rest of the body by hemoglobin in the blood. COVID-19 destroys this process causing oxygen debt, but this may be a secondary symptom. White blood cells release inflammatory molecules in response to abnormality in the cell leading to inflammation and apoptosis (cell programmed death). This is the underlying pathology of pneumonia, with its corresponding symptoms: coughing; fever; and rapid, shallow respiration. Some COVID-19 patients recover, sometimes with no more support than oxygen. Others deteriorate, often quite suddenly, developing a condition called acute respiratory distress syndrome (ARDS) (13). Oxygen levels in their blood plummet and they struggle ever harder to breathe (13, 14). On x-rays and computed tomography scans, their lungs are riddled with white plaques resembling ground glass. Commonly, these patients must be artificially ventilated and their mortality is high. There is no evidence that extracorporeal membrane oxygenation (ECMO) machines that bypass the lungs ameliorate the systemic sepsis and mortality rates of patients from those on a ventilator, probably because the oxygen carrying capacity of the blood is severely attenuated (14). Autopsies have shown that the alveoli were inundated with fluid, eukaryotic material and dead lung tissue (11, 12). Concurrent with this pathology organ failure renal, hepatic and cardiovascular are precipitated by sepsis of the capillary network (15). Subsequent prevalence of clotting, multiple organ failure and all the symptoms of sepsis give a diagnosis of systemic septic shock in almost all deaths.

Most individuals recover quickly from COVID-19 infection, producing antibodies to the disease. However, it should be noted that most patients who do not recover have already either begun to produce antibodies (11) and weak immune system is not a factor in their deaths, but a slow immune system can have fatal consequences. Almost all infected individuals survive the first infection of the virus. Virion levels rise to their maximum and almost all patients produce antibodies. The antibody response is therefore critical to recovery but does not necessarily preclude mortality. Timing is critical between the infection and antibody response and the vulnerabilities of an individual determine their survival.

## Bacterial vs. Viral Infection

The basic parameters of bacterial and viral infections differ. Bacteria use their own cellular structures to multiply and do not require the host cellular structures- they can use a primordial pool of material, viruses are restricted to using the host cells own structures to multiply and thus must retain the structural integrity of the host to continue to multiply. Once the cell is depleted of material capable of sustaining the ongoing multiplication deficits will occur disturbing the inherent makeup of cellular components rendering visible to the immune system. For cellular structures to remain intact a constant stream of trophic material must be provided to the cells. Unlike bacteria that can survive and proliferate within a dead environment, viruses like Sars-COVID-19 cannot. The early action of the virus is thus not to damage its own environment and damage to the cell is initially from molecular instability external to normal metabolism and a consequence of cellular logistical inefficiency to provide the requisite materials for the virus to continue its replication. Although often overlooked the virus in early stages of infection is more dependent upon its host than a bacterium. In turn the mechanism of reproduction and subsequent depletion is of is fundamentally important when considering the spread and infection of COVID-19 virions within a body and systemic release and functional damage and subsequent death. If the virion is permitted to replicate where the logistics of cellular structural integrity remains little change will come to the cell. Only when the material for reproduction in terms of molecular quantities of building materials ceases will the mRNA of the virion continue to consume the cellular structure of the host cell. This in turn may change the cellular membrane structure making it visible to antibodies.

## Apoptosis

The action of cytokine system (16, 17) is not to destroy cell in response to an infection but as a result of cellular incapacity to operate within normal limits as to its function (18). COVID-19 replication depletes the infected cell of material, and usurps its mechanisms. The change from normal activity in turn changes the biochemical nature of reactions at the molecular level starving necessary components necessary to maintain homeostatic integrity. Any action considered normal for the cell does not therefore produce apoptosis, but cytokine release to instigate apoptosis is a result of major cellular damage.

## Speed of Infection and False Immunity

Viral and host factors play important roles in the course of infection. Critical to life expectancy and prognosis is the speed of infection and subsequent immunity. The development of immunity is critical in the systemic spread of COVID-19 and some assumptions must be questioned as to their scientific acceptability. Immunity can either be from vaccination or from previous exposure. In both cases the body retains antibodies that deteriorate over time (15). Viruses can quickly mutate (19). A COVID-19 vaccination may be ineffective against any new mutation (20). In addition, vaccination against a specific virus will only work effectively while enough antibodies exist in the body with the ability to multiply at a fast enough rate to negate the disease (20). This will decrease with time and immunity will only occur if there are enough antibodies to be replicated fast enough to overcome the multiplication of virions before they reach the point where apotosis and sepsis takes place. The rules therefore are dependent upon how much each individual is immunosuppressed due to their inherent vulnerabilities and the infection rate. Antibody production does not occur instantly and individuals with immunity are therefore capable of spreading the disease during this process. Thus, all individuals, whether immunized or not, whether showing symptoms or not, become infectious as the immunity commences and this infection continues until antibodies have destroyed every virion present in the body. All individuals therefore are inherent carriers and spreaders of the disease, whether they have immunity or not, by either vaccination or previous infection. As yet, there is no evidence that either vaccination or prior exposure prevents a second exposure from being infectious during the time between infection and destruction of the virus.

In a bacterial infection bacteria divide doubling according to time and medium and occurs exponentially until resources for division are expended. Viruses by contrast do not require division to replicate but use the mRNA and the sarcoplasmic reticulum of the host cell. A single virion may infect a cell and its RNA produce many virion copies unrestricted by the requirement to divide. Virus replication may therefore occur faster than simple exponential division with a single virion multiplying many times up to the point where material for replication is expended. A single virion infecting a cell is therefore sufficient to replicate until the materials for its own division have been expended. Each cell infection also has a critical limit of replication caused by depletion of nutrients which lead to cell death.

## Acute Respiratory Distress Syndrome and Sepsis

ARDS occurs when fluid builds up in the alveoli and is associated with sepsis (21). Sepsis is life-threatening condition due to a dysregulated host response to infection, which is time-dependent and associated with unacceptably high mortality (13, 22). Mortality remains higher than 25–30% and even higher when shock is present. No effective specific anti-sepsis treatments exist (23).

Sepsis results when an infection triggers a localized inflammatory reaction that causes systemic symptoms of fever or hypothermia, tachycardia, tachypnoea, and either leucocytosis or leukopenia, i.e. the systemic inflammatory response syndrome (24). Severe sepsis is defined by dysfunction of one of the major organ systems. The inflammatory reaction is mediated by the release of cytokines. Cytokines activate the extrinsic coagulation cascade and inhibit fibrinolysis. According to Assiri et al. (25), higher concentrations of the pro-inflammatory cytokine interleukin-6 (26) and the fibrin degeneration product D-dimer (27) were strongly associated with in-hospital mortality in COVID-19. These overlapping processes result in microvascular thrombosis. Thrombosis is one potential factor producing organ dysfunction. Management of patients with sepsis relies mainly on early recognition and correct therapeutic measures. Treatments involve antibiotics resuscitation with intravenous fluids and drugs acting upon targets that are components of the inflammatory response, cytokines such as tumor necrosis factor (TNF), interleukin 1, interleukin 6, or platelet activating factor, or components of the coagulation cascade or vasoactive molecules (28).

The mechanism of sepsis is systemic, being intrinsic to the fluid and logistic mechanisms of circulation, with symptoms originating at the cellular level (29). This pathology manifests in the epithelial cells separating tissues from the external environment and capillary leak syndrome (30). Epithelial cells maintain structural integrity of tissues including the small capillary vessels that surround the alveoli in the lungs and the capillaries of all other organs. Epithelial cells are lined with a gel-like surface of interconnected proteins call the endothelial glycocalyx (31).

## Endothelial Glycocalyx Layer (EGL)

The endothelial glycocalyx layer has an essential role in maintaining the normal fluid homeostasis of the body (32), but is fragile and can be damaged by a number of pathophysiological conditions and interventions.

The physiology of the endothelial glycocalyx layer has been shown to significantly affect the microcirculation reducing fluidity in capillary flow. Recently the Starling principle of fluid reabsorption and oncotic maintenance in the capillary network has been shown to exist almost exclusively across this layer and not as previously supposed across the capillary system. This has produced the reformed Starling principle (31) that defines fluid distribution more accurately as occurring almost exclusively though this layer. The EGL is therefore essential in maintaining correct oncotic pressure in the capillary bed as well as influencing the adsorption and reabsorption across the capillary membranes. In fluid therapy where osmotic pressure changes, damage may be done to this layer decreasing its thickness and altering its fundamental structural integrity—this is well summarized by Kundra (31). Thus, the EGL maintains the functional integrity and mechanisms supporting not just the physical integrity of the endothelial transport system but also its pathophysiology. In (33) it was concluded that ‘Glycocalyx damage may be limited by avoiding hypervolemia and hyperglycaemia and by maintaining a physiological concentration of plasma protein, particularly albumin’. Plasma transfusion has been effective protection of the EGL (34) and has been implicated in preeclampsia (35). Albumin has been proposed for therapeutic targeting of the EGL (36). The main point is that under physiological conditions oncotic pressure is maintained by human serum albumin HSA.

In septic shock damage to the EGL can also cause inflammation of the vascular endothelium and maldistribution of microvascular blood flow as well as the release of the damaging free radical such as nitric oxide (37) which is normally involved in regulation of vascular homeostasis (38), but uncontrolled release is likely to add to tissue and erythrocyte damage, thus reducing the oxygen carrying capacity of blood. The current literature on serology in patients with COVID-19 have reported decreased hemoglobin concentration in these individuals (39). They stated that coronavirus binds to hemoglobin and frees the iron ions, leading to impairment in the oxygen delivery to the vital organs, release of free radicals and thus elevation in stress, oxidative load, hypoxia and finally heart attack or cardiac arrest may occur (40, 41). There is further evidence for hemoglobin impairments, such as elevated serum ferritin, erythrocyte sedimentation rate, C-reactive protein, lactate dehydrogenase (40, 42). Underlying all of this is damage to the EGL due to modulation of serum albumin by the SARS-CoV-2 virion-albumin binding site as explained below.

## Human Serum Albumin

Serum albumin (43) functions as a significant regulator of plasma oncotic pressure and a transporter of ligands (44–46). It has a half-life in the body of about 20 days and decreases by 10–15% after 50 years of age (47). Importantly 80% of free albumin is contained within the interstitial spaces where it must be assumed to perform the same transport function. Albumin is created in the liver which maintains its concentration, but this reduces after 50 years of age (47). In clinical medicine, serum albumin can be measured via standard serum laboratory testing that measures both free and bound albumin. Free fatty Acids (FFA), melatonin (2, 48–50) and the SARS-CoV-2 virions are transported bound to albumin as are glycolates. Studies on drug interactions confirm both competitive and non-competitive binding (49, 51–54). The role of albumin is well accepted in regulation of hormones (55–57). Research on albumin has provided new insights such as the characterization of the pleiotropic effects of albumin (58). Conformational change to albumin can affect binding of viruses (59). The exact binding sites and therefore the interactions between protein FFA are unknown however the basic mechanism of competitive and non-competitive binding sites have been described. Albumin binds, mostly non-specifically, with each ligand having different affinity for competing sites (60). Glycosylated albumin has long been known to affect platelet aggregation (61) a factor of clotting. Hypoalbuminemia is frequently observed in patients with conditions like diabetes, hypertension and chronic heart failure, and who are statistically most vulnerable to SARS-CoV-2 infection. According to Huang et al. (62) low albumin levels are seen in almost 81% of non -surviving COVID-19 patients and a clinical trial to examine the effects of intravenous infusion of albumin to COVID-19 patients with respiratory insufficiency has recently been registered in India (63). Recently hypoalbuminemia, coagulopathy and vascular disease have been linked in COVID-19 (64) and has been shown to predict outcome independent of age and morbidity (65). Hypoalbuminemia is a known factor in sepsis and ARDS (46, 66–68).

Albumin bonds to ligands by a reversible process where the equilibrium between bound and unbound depends upon the relative concentrations and depends upon ligand. All ligands capable of binding including proteins fatty acids and indeed the SARS-CoV-2 virus are bound in competition for sites to bind upon the albumin molecule as other viruses (45). There are both specific and non-specific areas of binding on an albumin molecule representing different affinities for different ligands (60, 69, 70).

## Binding of SARS-CoV-2 to Albumin

**Figure 1:**
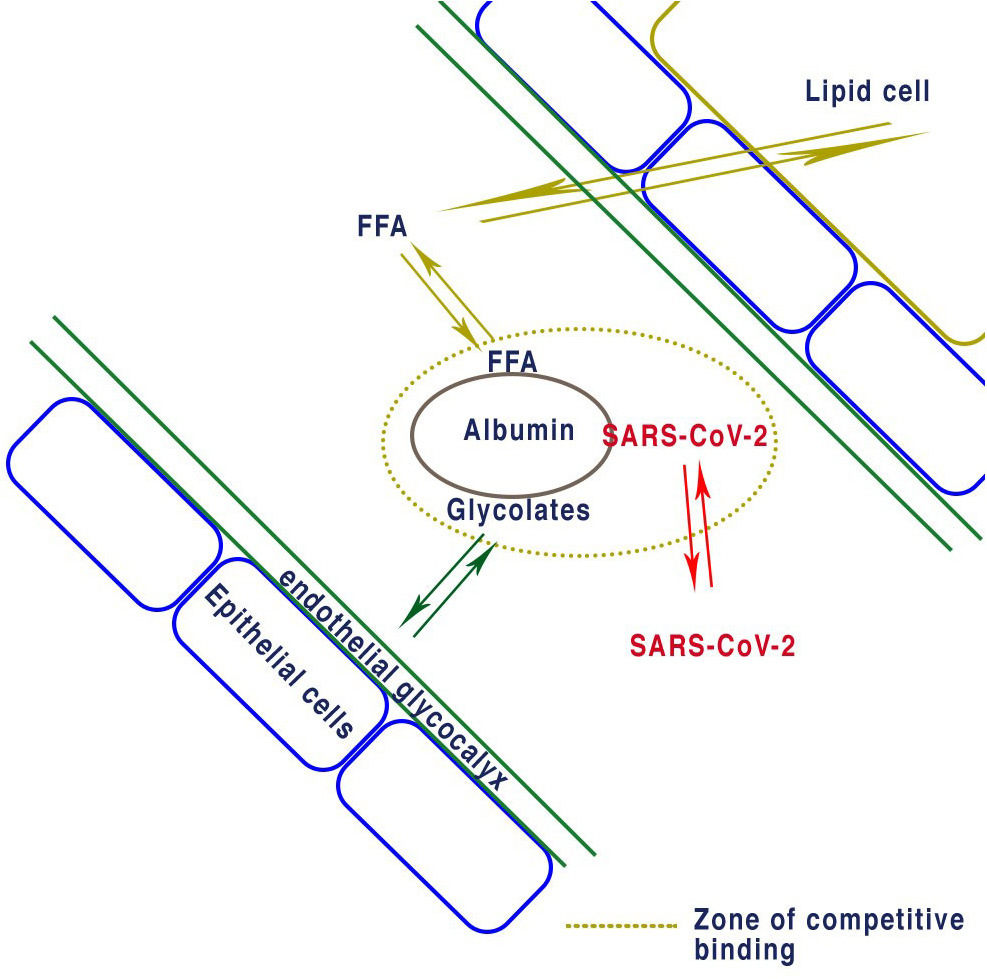
Proposed equilibrium of binding for albumin with FFA determined by lipid cells. Reversible albumin binding of both EGL proteins and FFA determines the concentrations non bound in plasma. If enough SARS-CoV-2 virions bind to albumin they will reduce the concentration of glycocalyx and thus deplete the endothelial glycocalyx layer. Similarly, excess FFA due to obesity causes reduction in the EDL or other ligands bound to albumin.

The nature of the SARS-CoV-2 virion-albumin binding site is unknown, but some permanent binding cannot be discounted as proposed in Figure 1. At the cellular level the SARS-CoV-2 virus enters the body and uses the cells own apparatus to replicate itself. One single virion can use the mechanisms inherently available to replicate itself in a single cell, and there is no evidence to suggest this process harms the cell. Damage to the cell can only be caused by instability of the cellular mechanisms by depletion of nutrients necessary for replication of the virus and the wellbeing of the cell. Depletion is most likely to occur by expenditure within the cell due to repeated virion manufacture and ejection. The result is an unstable cell vulnerable to apoptosis. The maintenance of the cell is the critical factor and is determined by the delivery of nutrients and the rate they are used. The levels of nutrients supplied by the blood determine this equilibrium.

Spread can be local where one cell releases directly to another adjacent cell or through the extracellular fluid surrounding the tissues leading to systemic release. Initially virions released from a site infection may remain localized. Once they are released, they travel in the blood and lymph bound to albumin.

The immunity conferred on the young must be because of the difference in the systemic environment. Obesity results in a rise in the plasma free fatty acids that are bound to albumin thus reducing the unbound portion (71). In COVID-19 patients, the level of serum albumin is decreased and the body requires more serum albumin (42). In cases of influenza and other respiratory viruses supplementation with bovine serum albumin was used as a transport medium in a community-wide surveillance of febrile respiratory disease for influenza viruses in order to attenuate the enhanced production of reactive oxygen species such as hydroxyl radicals by neutrophils during an influenza viral infection (72, 73).

## Conclusion

Unlike influenza where both young and old were affected SARS-CoV-2 mortality increases over 50 years of age. There are very few directly measurable systemic events that change during aging that could be a culprit for death occurring among the over 50's and obese. The reduction of the overall concentration of albumin in the extracellular body fluids is the most likely source of limitation for the transport of substances around the body transporting up to 80% substances. Here we suggest that the albumin complex is inherently the maintainer of homeostatic regulation of essential cellular nutrients as well as oncotic pressure. Our model suggests that albumin transport plays a direct role in the stasis of nutrients that supply the cellular structures that regulate the thickness of the EGL. This provides a direct link from the behavior of cellular SARS-CoV-2 infection, in this case to the destruction of this layer leading to sepsis and general septic shock.

On infection the initial response of infected tissues is to accommodate the virus. This is accompanied by a release of virions into the systemic system where they are bound in equilibrium with Human serum albumin. As infection continues and viral particles expand the ratio between bound and unbound virion-albumin increases until a crisis point is reached. The bound albumin-virion displaces the ligands present on albumin by corresponding amounts. Reduced nutrients provoke cell stress and then apoptosis. Tolerance to the virus thus depends upon, amount of free-albumin. As infection continues the ability of albumin to transport nutrients depends upon the extent of free albumin. Albumin moderates binding the levels of nutrients in the blood. Release of virions into the bloodstream therefore prevents albumin from transporting nutrients into the cells.

We conclude therefore that albumin therapy to replace bound albumin, or strategic addition of HSA to increase the overall concentration in the fluid components of delivery (plasma and interstitial fluid, etc.) might alleviate some of the symptoms leading to sepsis (23, 74). A rise to average albumin levels we predict could alleviate systemic sepsis and prevent death (23, 74). Fluid therapy bundles are available (75–77) containing albumin. However, these are of very low percentages 4% which may become quickly bound and a more long-term sustained approach would be preferable (78).

Fluid therapy is especially important in many surgical situations and this type of physiological intervention whilst not commercially attractive is none the less extremely valid and has a vast history of solid physiological and biochemical research evidence to support it. This is an area of research that is important and apparent in every operation where fluid is given has historically been neglected.

By changing the albumin-bound to albumin-free systemic stress can be reduced with the hope that more lives are saved.

## Author Contributions

The basic concept was suggested by AJ. AJ and WW worked on the manuscript together with very useful inputs and suggestions from RF. All authors contributed to the article and approved the submitted version.

## Conflict of Interest

The authors declare that the research was conducted in the absence of any commercial or financial relationships that could be construed as a potential conflict of interest.
